# Patient Empowerment Using Electronic Telemonitoring With Telephone Support in the Transition to Insulin Therapy in Adults With Type 2 Diabetes: Observational, Pre-Post, Mixed Methods Study

**DOI:** 10.2196/16161

**Published:** 2020-05-14

**Authors:** Helen McGloin, Dympna O'Connell, Michele Glacken, Patsy Mc Sharry, Denise Healy, Lisa Winters-O'Donnell, Kathleen Crerand, Anne Gavaghan, Louise Doherty

**Affiliations:** 1 Department of Nursing, Health Science and Disability Studies St Angela's College Sligo Ireland; 2 Institute of Technology Sligo Ireland; 3 HSE West Letterkenny, Donegal Ireland

**Keywords:** diabetes mellitus, type 2, telehealth, insulin, empowerment, self-management

## Abstract

**Background:**

Initiation of insulin therapy for the management of type 2 diabetes can be an unwelcome and distressful development for patients. Current evidence suggests that telemonitoring can help improve glycemic control in type 2 diabetes and can support empowerment to self-manage diabetes. This telemonitoring intervention was underpinned by an empowerment approach.

**Objective:**

This study aimed to evaluate the clinical effectiveness and feasibility and the patients’ and health care providers’ experiences of a 12-week telemonitoring intervention with telephone support for patients commencing insulin therapy. This paper focuses on the impact on patient empowerment.

**Methods:**

An observational, pre-post, multimethod, and triangulation design was employed to study a 12-week automated electronic telemonitoring intervention with telephone support from a diabetes clinical nurse specialist (CNS). Forty patients were recruited from the clinic as they were about to commence insulin therapy. In the quantitative arm, biometric data (hemoglobin A_1c_ [HbA_1c_] and weight) and psychosocial data (diabetes empowerment scale [DES] scores and diabetes distress scale [DDS] scores) were gathered by the research team at baseline (T1), the end of the intervention (T2), and 3 months postintervention (T3). Data on hospital admission and general practitioner (GP) visits were collected for the duration of the study. In the qualitative arm, separate focus group interviews were conducted with the CNS team supporting the intervention (n=2) and patients (n=16).

**Results:**

Of 39 patients who completed the intervention, 23 (59%) were male. The mean age of the sample was 62.4 years (range 37-80 years). The mean HbA_1c_ (mmol/mol) decreased significantly between T1 and T2 (mean difference [MD] −17.13; *P*<.001) and T1 and T3 (MD −18.16; *P*<.001), with no significant impact on weight. In the focus groups, patients reported an increased awareness to self-manage diabetes and feelings of safety and comfort. There were 13% (5/39) of patients who had hypoglycemia on two or more occasions. A significant increase in the mean DES score occurred between T1 and T2 (MD 0.62; *P*=.001) and T1 and T3 (MD 0.72; *P*<.001). The mean DDS score decreased between T1 and T2 (MD −0.64; *P*=.002) and T1 and T3 (MD −0.6; *P*=.002). The mean patient satisfaction with the intervention was above 4 out of possible 5 on all items on the Telemedicine Satisfaction and Usefulness Questionnaire. We observed a reduction in diabetes clinic attendances and GP visits. A significant increase in workload was reported by the CNS team.

**Conclusions:**

This intervention had an empowering effect for patients in the self-management of type 2 diabetes and has the potential to meet the need for safer and more effective care in insulin initiation in the community setting. We observed a significant increase in workload for health care staff. Telemonitoring needs to be streamlined with health care delivery and accompanied by adequate support services.

## Introduction

### Background

The goal of diabetes treatment is optimal glycemic control and prevention of complications. International guidelines recommend the initiation of insulin when people with type 2 diabetes have signs and symptoms of acute decompensation that are no longer controlled by oral hypoglycemic drugs and lifestyle [1]. Insulin initiation is often delayed, and once started, achieving optimal doses requires a frequent scheduled review of blood glucose levels and individualized dose titration [2]. The failure to commence and intensify treatment is termed *clinical inertia* and is a considerable problem in primary care practice [3]. There are multiple barriers to optimal insulin treatment in primary care at the patient and practitioner levels, and there is a need for interventions to improve self-management support and integrated insulin support systems [4].

Once insulin is commenced, many patients are not followed up until the next clinic appointment, resulting in delays in achieving the optimal insulin dosage [5]. The barriers to initiating insulin include misconceptions about insulin, perceived difficulty in management for both physicians and patients, and risk of hypoglycemia and weight gain [6]. Lack of time is a common health care system–related barrier to insulin initiation [5]. Starting insulin is stressful for patients, and providing adequate support and monitoring during this process can present challenges for health care providers, particularly in between health care visits [7]. The safe and effective transition to insulin therapy requires health care providers to examine alternative ways of empowering the patient in self-management.

Empowerment can be viewed as both a process and an outcome [8]. It can be viewed as a process when an intervention aims to equip patients and their families with the self-awareness, autonomy, knowledge, and skills to become *comanagers* of their condition in partnership with health professionals. The desired outcome is enhanced confidence and skills to manage the physical, emotional, and social impacts of diabetes in their daily lives. Patients who feel empowered by their health care practitioner develop a greater sense of self-control, which may lead to better glycemic control [9]. Positive effects of telemonitoring include patient empowerment to self-manage [10] healthy coping and problem solving [11], and improved practitioner-patient relationships and patient engagement have also been reported [10-13].

A review by Risling et al [14] found that the consistent lack of conceptual clarity on what constitutes empowerment in the electronic health (eHealth) context means that evaluating patient empowerment associated with eHealth technology is challenging. More recently, Risling et al [15] found that patients in a digital study identified the relational and informational elements of empowerment and recommended that these key areas should shape the focus of the evolution of patient empowerment in digital research. Telemonitoring has been found to be effective as a confidence, decision-making, and self-care enhancer in older persons with chronic heart failure [16,17]. Telemonitoring surveillance systems have left patients with feelings of greater self-control, heightened motivation for lifestyle changes, and improved quality of life [18].

### Aims

**Figure 1:**
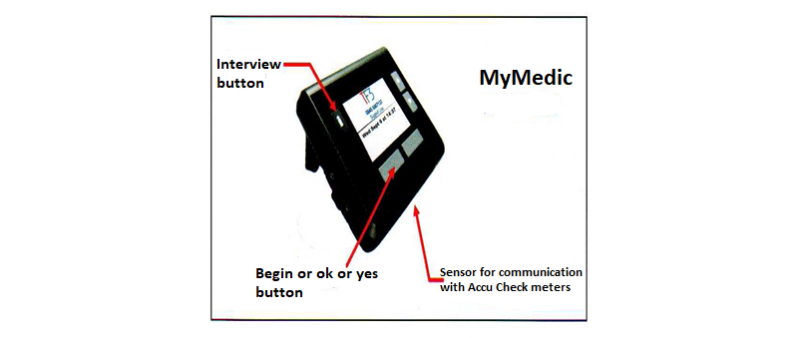
MyMedic hub.

This study sought to implement and evaluate telemonitoring with telephone support in a real-world setting using an observational, pre-post, mixed methods design in a cohort of patients who were about to commence insulin therapy. A MyMedic hub (Figure 1) was placed in the patients’ home for 12 weeks. The patients recorded their blood glucose readings as normal, and at the times agreed, they sent their blood glucose readings to a monitoring center using the hub. The schedule for upload was agreed between the diabetes team and the patients, and the hub unit prompted the individual to upload their readings as agreed. The team reviewed the blood glucose results and contacted the participant, if necessary, to seek additional information on their symptoms and well-being. Insulin was adjusted according to need via telephone discussions using an insulin adjustment plan.

This paper aimed to describe the impact on hemoglobin A_1c_ (HbA_1c_), hypoglycemic events, patient empowerment, diabetes distress, and satisfaction with telemonitoring from the patients’ perspective. In addition, the experience of using telemonitoring to facilitate the transition to insulin therapy was explored from the perspective of the diabetes team and health care organization.

## Methods

### Study Design

An observational, pre-post, multimethod, triangulation design was used to evaluate the feasibility, clinical effectiveness, and resource implications of telemonitoring support for patients commencing insulin therapy in the real-world setting. The design was underpinned by an empowerment philosophy. The mixed methods approach allowed for a holistic overview of using telemonitoring in diabetes care, generating physiological data and exploring receptiveness of technology and the core aspects of empowerment.

### Population

To be eligible for inclusion in the study, patients had to be aged older than 18 years with type 2 diabetes and commencing with insulin therapy. Patients with a score below 4 on the clock drawing test were excluded, as this indicates the cognitive inability to self-manage insulin [19]. The patients were recruited by the clinical nurse specialist (CNS) team from hospitals and community-based diabetes clinics in the Northwest of Ireland. The sample size was determined by cost, available time of the CNS to support, telemonitoring equipment, and timeline for the feasibility study resources to provide the intervention. The project team determined that 40 patients would be enough to yield useful data and be achievable within the available resources to fund the project.

### Sample

Convenience sampling was used. When the decision to commence a patient on insulin was made, the CNS invited them to participate in the study. One participant chose to discontinue from the study after 1 week because of the problems experienced with connectivity. The recruitment took place from April 2016 to June 2017.

### Intervention

Following recruitment and consent, the patients were commenced on insulin therapy and provided with the telemonitoring system in addition to standard care. Standard care of this cohort includes telephone calls with the CNS with visits to the diabetes clinic as needed to collate blood glucose readings and to adjust insulin levels once a week.

A MyMedic hub (Figure 1) was placed in the patients’ home for 12 weeks by a telecare support officer from Fold TeleCare who was contracted to provide the monitoring on behalf of the Health Service Executive (HSE), Ireland. The telecare support officer who installed the hub in the home taught the patients how to send their readings and gave instructions about what to do if anything did not work.

The patients recorded their blood glucose readings as instructed by the CNS. The usual practice is that patients on basal insulin self-test their blood glucose levels four times daily or more to establish the pattern of glucose through the day and to comply with the HSE guidelines [20]. At the times agreed, they sent their blood glucose readings to a monitoring center using the hub. The hub unit prompted patients to upload their readings as agreed with the CNS. Most patients uploaded twice a week for the first 3 weeks and then weekly for the remaining 9 weeks, as they needed more intensive support at the beginning. This was based on a similar study by Turner et al [21]. We contacted the authors to find out about their titration guide and frequency of dose adjustment, which was every 3 days initially and then weekly once stable. The American Diabetes Association and the European Association for the Study of Diabetes joint guidelines [22] recommend increasing basal insulin dose by 2 IU every 3 days until fasting glucose reaches the desired range individualized for each patient. As the target is neared, dosage adjustments should be more modest and occur less frequently.

None of the patients required more than weekly uploads after week 3. If the participants did not upload their blood glucose levels at the scheduled time, the telecare support officer contacted them by telephone to remind them.

**Figure 2:**
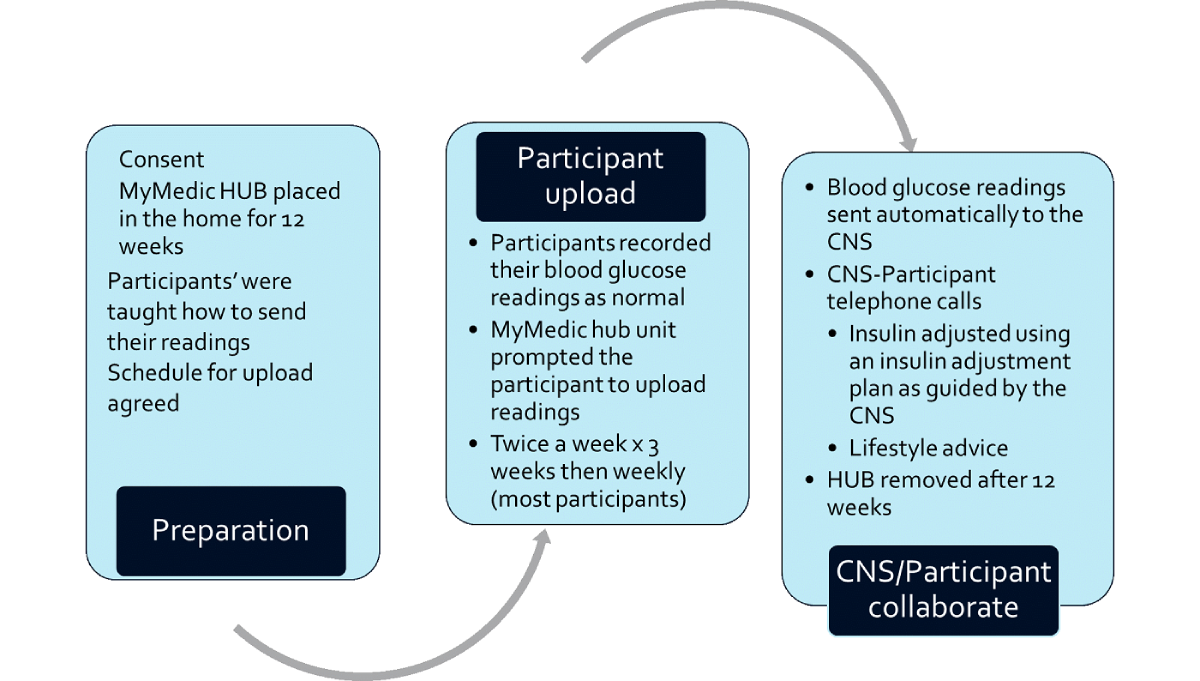
Intervention. CNS: clinical nurse specialist.

The readings were sent automatically to the web-based platform. The CNS accessed the data for the individual on a prearranged date and contacted the participant, if necessary, to seek additional information on their symptoms and well-being. The record was displayed in a diary format, showing the patient’s readings at each time of the day. This allowed the review of glucose data throughout the day. Charts, graphs, and tables could be created from these data to allow analysis. Insulin doses were entered onto the system with any changes to the care plan to allow sharing of data between health professionals. Blood glucose data were saved onto the patients’ health service electronic file in pdf. Patients could contact their CNS or general practitioner (GP) if they were worried about their blood glucose readings, as per usual care. Insulin was adjusted according to need using an insulin adjustment plan, as guided by the CNS. After the 12-week period, the hub was removed from the patients’ home (Figure 2).

### Usual Care

A retrospective audit of 12 patients from 2015 showed that clinic visits ranged from 1 to 3 (mean 1.75) in the 12-month period following the commencement of insulin therapy. Phone calls from the CNS ranged from 0 to 4 per patient (mean 1.7). In this cohort, the mean HbA_1c_ remained relatively stable over 3 collection points—at baseline (T1; 8.5%), 6-month follow-up (8.6%), and 12-month follow-up (8.4%). No clinically or statistically significant changes occurred across the time points.

### Data Collection

Data on biomedical variables were collected at a face-to-face meeting with the CNS. HbA_1c_ and BMI data were collected at T1, 12 weeks (at the end of the intervention; T2), and 6 months postbaseline (3 months postintervention; T3). The CNS kept an ongoing telephone call log of insulin dose, patient-reported hypoglycemia symptoms, call frequency and length, and main topics discussed in the calls. In addition, self-efficacy was measured using the Diabetes Empowerment Scale-Short Form [23], and the Diabetes Distress Scale was used to assess diabetes-related emotional distress [24], at the same time points. Satisfaction with the telemonitoring intervention was measured using the Telemedicine Satisfaction and Usefulness Questionnaire at T2 [25]. All T2 and T3 questionnaires were completed remotely via telephone by a researcher not involved in the intervention. All data were entered into the SPSS statistics version 24 (IBM Corp) and checked for accuracy.

All telemonitoring patients were invited to a focus group interview at T2. Two focus group interviews were conducted with 4 and 12 patients in the first and second groups, respectively, at a community health care venue. The aim of each interview was to explore the expectations, achievements, and opinions of the patients of the telemonitoring intervention, and the interviews were facilitated by an experienced interviewer not involved in the intervention, using a topic guide. Once all patients had completed the intervention, the 2 diabetes nurse specialists who coordinated the intervention were interviewed.

### Data Analysis

#### Quantitative Data

Tests of distribution were carried out on all variables before statistical analysis. Variables with a normal distribution were analyzed using the repeated measures analyses of variance test to compare the pre- and postintervention HbA_1c_, weight, BMI, diabetes empowerment scale (DES) score, and diabetes distress scale (DDS) score at T1, T2, and T3. A *P* value of less than .05 was considered significant. Variables not normally distributed were analyzed using the Friedman test, and if significant, the Wilcoxon test was used to determine which time points are significantly different from T1.

#### Qualitative Data

Initially, all interviews were openly coded. The descriptive content analysis was carried out using a framework [26]. Emerging codes were examined and compared for any overlap in meaning, and similar codes were collapsed to form higher-order codes. This constant comparative process continued throughout the data analysis process until the major categories that account for the data were developed. The relationships between categories were explored and made explicit. During the analytic process, detailed memos were recorded, which tracked the emerging understandings and the relationship between the categories identified. The findings are reported textually, supported by relevant quotations from the participants. The computer software package NVivo 10 (QSR International) was used to assist in the organization, management, and retrieval of the qualitative data.

### Ethics

Ethical approval was received from the regional hospital Research Ethics Committee before commencement of the study. Mechanisms were put in place to ensure that the rights of the participants and their well-being were given precedence over data collection. All proposed participants were given both written and verbal information about the study. Patients were informed about the study face to face and invited to participate. Willing patients were met by the CNS to obtain written consent to participate. All data were anonymized and password protected and stored in accordance with the Data Protection Act, Ireland [27].

## Results

### Demographics

A convenience sample of 40 patients commenced the intervention, with 1 participant withdrawing early because of connectivity issues. All remaining 39 patients completed the 12-week telemonitoring and completed data collection for all time points postintervention. Table 1 provides the participants’ demographic information.

**Table 1:** Participant demographics (N=39).

Demographics		Values
**Age (years)**		
	Mean	62.4
	Range	37-80
**Gender, n (%)**		
	Male	23 (59)
	Female	17 (43)

### Biomedical Variables, Hemoglobin A_1c_, Weight, and Insulin Dose

**Figure 3:**
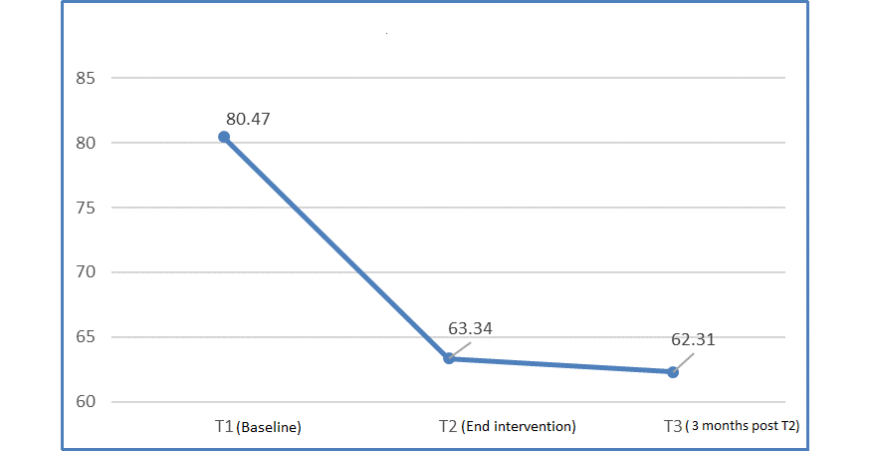
Changes in mean hemoglobin A_1c_ (mmol/L).

**Figure 4:**
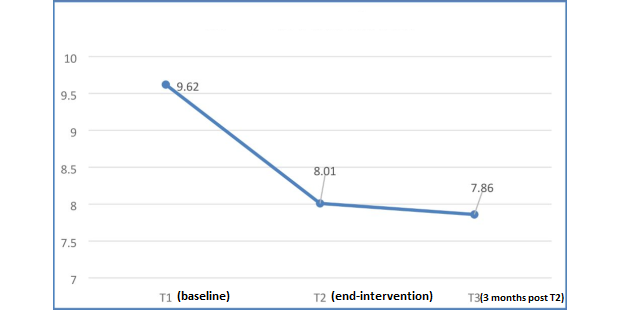
Changes in mean hemoglobin A_1c_ (%).

HbA_1c_ (mmol/mol) decreased significantly between T1 and T2 (mean difference [MD] −17.13; *P<*.001) and T1 and T3 (MD −18.16; *P<*.001). This represents a 21.2% drop in HbA_1c_ at T3 (Figures 3 and 4). No significant change occurred in weight or BMI over the 6-month period (Table 2). The mean insulin dose at T1 was 17.25 IU (SD 11.1) and ranged from 6 to 60 IU. We audited HbA_1c_ at follow-up in clinic visits in 2019 and found that the changes in HbA_1c_ (mean 60.4 mmol; *P*<.001) from T1 were maintained.

**Table 2:** Change in weight and BMI among participants (N=39).

Timepoint	Weight (kg), mean (SD)	BMI (kg/m^2^), mean (SD)
T1 (baseline)	85.21 (22.75)	30.16 (7.32)
T2 (end of the intervention)	85.18 (20.93)	30.15 (6.82)
T3 (3 months postintervention)	85.63 (21.55)	30.36 (6.97)

### Diabetes Empowerment Scale Scores and Diabetes Distress Scale Scores

**Figure 5:**
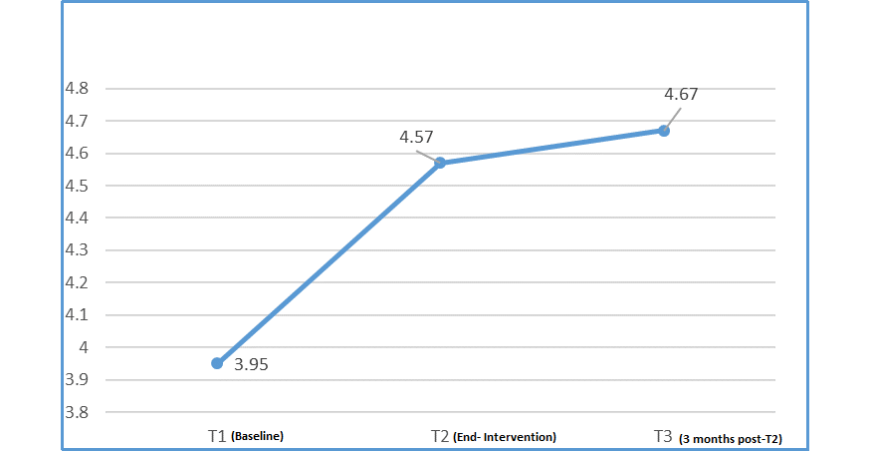
Changes in mean total diabetes empowerment scale (DES) scores.

**Figure 6:**
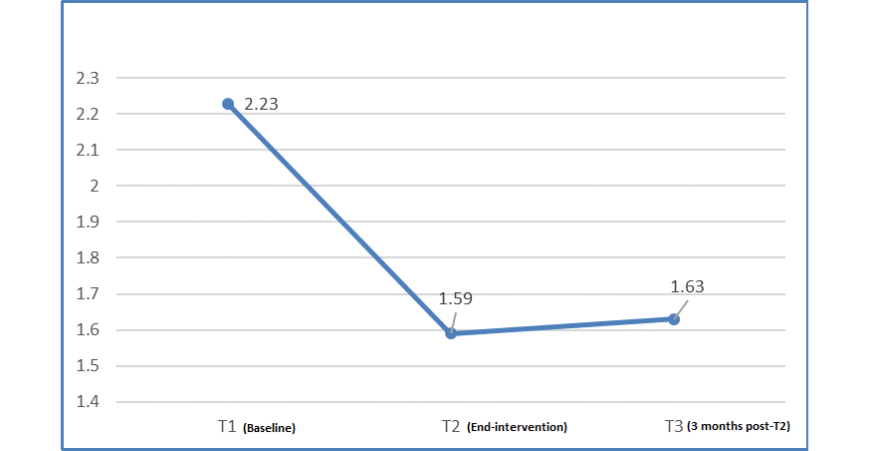
Changes in mean total diabetes distress scale (DDS) scores.

From T1 to T2, an increase in mean DES score (MD 0.62; *P<*.001) and a decrease in mean DDS score (MD −0.64; *P*=.002) occurred. These differences from T1 were maintained at follow-up at T3 (DES score: MD 0.72; *P<*.001 and DDS score: MD −0.6; *P=*.002; Figures 5 and 6).

### Hypoglycemia

A total of 13% (5/39) of patients had low blood glucose levels during the intervention. Specifically, 3 patients had 2 to 3 episodes, which stopped with advice or dose adjustment. The other 2 patients had multiple episodes, resulting in one patient discontinuing insulin and the other having insulin doses adjusted several times during the study and after. However, during the telephone calls with the CNS, 38% (15/39) of patients reported symptoms of hypoglycemia, demonstrating the need for support for people commencing insulin therapy.

### Patient Satisfaction

The mean satisfaction score was above 4 out of a possible 5 on all items on the Telemedicine Satisfaction and Usefulness Questionnaire, indicating a high level of participant satisfaction with the telemonitoring intervention.

### Timing and Frequency of Telephone Support

The number and duration of calls were tailored to patients’ need. The mean number of calls logged during and after the intervention per participant was 11.5 (SD 4.16). The mean length of the call per participant ranged from 4 min to 15 min. The majority (369/450, 82.0%) of calls were initiated by the CNS, 11.6% (52/450) of calls were initiated by the patients, and 6.2% (28/450) initiation was not documented. The calls reduced dramatically on completion of the intervention, accounting for 8.0% (36/450) after 12 weeks.

The most frequent issue discussed in the telephone calls was poor glucose control (n=176) experienced by the majority of participants (36/39, 92%), followed by injection technique (n=76) reported by 92% (36/39) of participants, patient-reported symptoms of hypoglycemia reported by 38% (15/39) of participants, and self-monitoring of blood glucose technique (n=43) reported by 74% (29/39) of participants. Insulin dosage adjustment (n=147) was the most frequent intervention given to participants (n=37) on one or more occasions by the CNS, followed by healthy eating advice (n=109) and physical activity advice (n=62).

### Hospital, General Practitioner, and Diabetes Clinic Visits

Of the total participants, 25% (10/39) attended the diabetic clinic and/or visited the GP and/or experienced an unplanned hospital visit. Of those, 18% (7/39) of participants attended the diabetes clinic, with 2 participants attending twice, and 10% (4/39) of participants visited the GP regarding their diabetes, with 1 participant visiting twice. Unplanned hospital admission was experienced by a minority (3/39, 7%) of the overall number of participants. In the retrospective audit of 12 patients who commenced insulin in 2015, there were 21 clinic visits—we do not have data for this groups’ GP visits or hospital admissions.

### Qualitative Findings

The thematic analysis of the patient focus group interviews led to 4 main themes: psychological impact of diabetes, increased diabetes empowerment, nurse in the corner, and using the technology. These and their associated subthemes are summarized in Multimedia Appendix 1. The CNS focus group resulted in 5 themes and subthemes, which are summarized in Multimedia Appendix 2: usual transition to insulin, safe transition to insulin therapy using telemonitoring, increased patient empowerment, administrative supports and requirements, and technology. For the focus of this paper, we have selected the themes that relate to patient empowerment for discussion and triangulated the findings from the patient and CNS focus groups and the questionnaire data.

#### Psychological Impact of Diabetes

In the focus groups, patients talked about the impact of diabetes on their psychological well-being and the need for more support for dealing with the emotions and stress experienced because of the diagnosis and the effects of the illness. They discussed the negative emotions experienced by them because of diabetes, including anger and agitation with the diagnosis and frustration with the complexity of managing diabetes:

There’s a lot of agitation and stress around it. For me, in the beginning...when I was diagnosed with diabetes I was very angry because I neither drank nor smoked and I always kept active and working.

#### Diabetes Empowerment

In the focus groups, the patients discussed increased self-awareness around the need to self-manage the chronic disease along with developing the knowledge, skills, and confidence to do so. Taking part in the telemonitoring project changed patients’ thinking around taking responsibility to manage their diabetes. Having the machine in their home and being responsible for monitoring the blood glucose shifted their thinking:

It changes your life forever. It’s up to yourself then—do you want it or not. You grab it with both hands.

Being accountable to someone was a motivating factor in increasing awareness and led them to consider and think about the results they were seeing:

It’s made me more aware anyway because I’m thinking all the time maybe these bloods are too high, and the nurse is going to say this or maybe they’re too low.

Patients’ distress decreased and confidence increased with the comfort of knowing that someone was keeping an eye on the blood glucose all the time:

It’s giving you control back. You’re getting some control over your diabetes when that thing is in the house. Like you say, you can go and download. If you don’t feel right about anything in the week that you have done it or in the couple of days, you can go and download it and the nurse will phone you back and reassure you whether it’s right or wrong or what you want to do. That’s what I like about it.

Another major source of self-efficacy was mastery experience through taking steps to control diabetes and seeing positive results. In this study, achieving blood glucose control increased their perseverance with managing diabetes:

You stick at it. If you’re getting good results all the time, you stick at it.

The patient focus group findings were corroborated by the data from the DES score questionnaires. A significant increase in mean DES score occurred between T1 and T2 (MD 0.62; *P=*.001) and T1 and T3 (MD 0.72; *P*<.001; Figure 4).

The CNS team focus group findings also support improved patient self-management with increased patient knowledge and confidence:

They’re now very confident in managing their insulin so it doesn’t stress them if the doctor says we’ll just increase that by 2 units. They usually know, and they’ll come out and they say to me, I knew we needed to increase that. So, they have gained great confidence in insulin which doesn’t happen to everybody.

The CNS reported problems in the past with the timely uptitration of insulin. Many barriers exist in the usual care of patients to achieving an optimal insulin dose because of not having access to complete and accurate patient data, large caseloads, and insufficient time for the practitioners to frequently contact the patients to adjust their insulin doses. Moreover, 14 patients effectively used an insulin self-adjustment tool, which resulted in earlier titration of insulin to gain timely and improved blood glucose control:

I just saw one of them yesterday and she said to me, I did put the insulin dose down a little bit a couple of months ago but actually I had to put it back up again, I didn’t ring you because I knew that would be fine...this is a 78-year-old lady. When I say she didn’t want to go on insulin, she was absolutely definitely against insulin. To just hear her talking so confidently about her insulin dose and adjusting it and the rationale for the changes she made was great actually.

Not everyone in the study was taught to self-manage insulin, as it was different from traditional practice and would require further exploration and development for implementation to the wider diabetes population. However, those patients who did use the tool demonstrated increased knowledge and a desire to titrate their own insulin in response to higher blood glucose levels:

If it went back up again it would be a case of going to the diabetic nurse and readjusting your insulin. I wouldn’t mind if I had to adjust the insulin from now on because you know how it works now.

#### Nurse in the Corner

Patients described the telemonitoring as “It’s like a nurse in the corner.” Having access to the diabetes nurses and knowing that they were reviewing their blood glucose levels gave the patients comfort and a sense of safeness. It also meant that there was quicker intervention when their blood glucose levels were not right, and in their opinion, it reduced their need to visit the hospital and GP.

The added comfort or security relates to the vulnerability the patients felt while commencing insulin. This was an unknown territory, and having close monitoring with professional oversight meant that they felt safe. This links to the development of confidence to manage their diabetes, as an improved emotional state underpins the development of self-efficacy or confidence to manage diabetes:

Having it there, I know it’s there and I know if I have a problem, I’m going to get the call and the nurse is going to talk me through it. It’s hard to explain the comfort that you have in that.

#### An Empowering Nurse-Patient Relationship

In the focus group, the CNS participants identified the need for a more equal relationship and enhanced partnership between the nurse and the patient and recognized the role of the intervention in empowering patients toward self-management. This eased the nurse into the watchful observer role:

I suppose that we are always talking about self-management and for the self-management to work it has to be teamwork. It can’t be a them and us. You’re always trying to build up relationships.

The CNS perceived that the change in the nurse-patient relationship was toward one of enhanced partnership—a mutual goal that allowed the patient and the nurse to have an equal footing in its achievement:

even after the telemonitoring period, we still get phone calls from the patients but it’s interesting that there’s more an equal relationship with us. It isn’t the kind of traditional nurse and patient relationship.

#### Increased Workload

CNS participants did highlight that although the telemonitoring system potentially led to much greater efficiencies in terms of patient monitoring and treatment, they strongly voiced the need to have adequate resources to support such a system. One of the key areas of concern was related to adequate nursing resources. As this new telemonitoring system generated large dataset for each patient and these data were being uploaded daily, there was an expectation that nurses would be seeing and reviewing these data regularly. However, because of workload pressures and staff resources, CNS participants reported that often patient data may not be reviewed for several days after upload, and they identified this as a source of concern for patient safety. They would recommend using the track, trend, and triage service, which was not used in this study:

the ‘buts’ are that it definitely added to our work time and I suppose that just the pressure of knowing that you had a responsibility to those patients to look at those readings no matter what else was going on in the service was an additional pressure, I think you would have to restrict the numbers, especially with just the 2 of us; you would have to decide on a certain number at each time. You couldn’t just do it all.

## Discussion

### Principal Findings

In summary, we observed a significant reduction in HbA_1c_ levels without a significant increase in weight. Patient empowerment scores increased and DDS scores reduced, and these findings were corroborated by the participants’ experiences explored in the focus group interviews. These effects were maintained at follow-up 3 months after the intervention ended. Other effects reported by the participants included increased knowledge and competence to self-manage their condition. Some participants engaged in self-titration of insulin using a tool developed by the CNS. A total of 5 patients had episodes of hypoglycemia. A significant increase in workload was reported by the CNS team, which led to several recommendations for streamlining the delivery of telemonitoring with the current service and for additional supports to the health care team.

### Patient Empowerment

Patient empowerment in diabetes is fundamental to achieving behavior change, and it is important that the motivation to change is driven internally rather than externally [8]. Self-determination theory proposes that addressing the 3 psychological needs—autonomy, competence, and relatedness—fosters a motivation to engage in healthy behaviors. The extent to which these 3 needs are met or unmet in the social context predicts well-being and thriving [28]. The findings of this study suggest that these 3 psychological needs were met. The CNS observed a positive impact on the nurse-patient relationship, with a balancing out of power and a sense of letting go from the CNS perspective. The perception of increased partnership was empowering to both parties. Patients’ autonomy increased with an improved awareness and a heightened level of responsibility for the self-management of diabetes. In a large prospective observational study involving 4341 multinational patients, the quality of the patient-provider relationship was significantly correlated with insulin adherence and HbA_1c_ levels [29].

The patients’ knowledge and competence increased in both the effect of their lifestyle behaviors on blood glucose control and how to change them. They attributed this change to the increase in the monitoring of their blood glucose level and the support from the CNS. In a review of qualitative data from seven trials and observational studies of telemonitoring for long-term conditions in primary care, Hanley et al [30] concluded that generating and recording the telemonitoring data had an empowering effect on patients to self-manage. Being active participants in their care increased confidence and enabled them to negotiate prompt access to care.

### Insulin Self-Management

Although the central aim of this study was to explore the experience of using telemonitoring to facilitate the transition to insulin therapy, some patients expressed confidence in their knowledge and skill to self-manage their insulin dosage. The CNS participants also indicated an increase in their confidence to let patients self-manage. Empowerment occurs when the goal of the health care practitioner is to enable the patient to critically think and make informed decisions about their care [8]. Self-titration of insulin is well established in type 1 diabetes, but for most patients with type 2 diabetes, dose titration is still carried out by physicians and diabetes nurse specialists. The evidence suggests that this is not the best process to achieve the optimal glycemic control, and self-titration of insulin in type 2 diabetes may be more effective [2]. Computer-assisted self-titration has been found to improve patient awareness of blood glucose management and increase self-efficacy to manage insulin [31]. Our findings suggest that the use of telemonitoring combined with self-titration of insulin empowers people to self-manage and warrants further investigation.

### Less Distress, Increased Empowerment Scores, and Fewer Attendances

High levels of diabetes distress negatively affect insulin adherence and glycemic control [29]. The DES scores and DDS scores demonstrated that patient confidence in the ability to manage diabetes increased and diabetes distress decreased significantly by the end of the 12-week intervention. These findings were supported by the results of the focus group interviews where patients and the CNS team spoke at length about reduced distress and increased patient confidence in their ability to self-manage diabetes. They also reported fewer attendances to the GP and hospital clinics, and we observed a reduction in the number of clinic visits for the intervention group compared with a retrospective cohort. However, the intervention group received a much higher number of calls from the CNS than the retrospective cohort. Telemonitoring and support for people with diabetes have previously demonstrated improvements in self-management and reductions in psychological distress [32].

### Glycemic Control

There was a clinically and statistically significant change in HbA_1c_ at 3 months after the end of the telemonitoring intervention to support the introduction of insulin in glucose management, however, with no significant impact on weight. HbA_1c_ reduced by 1.61% at T2 and 1.76% at 6 months from T1. It could be argued that this reduction would usually be observed with the introduction of insulin, and without a control group, it is difficult to posit that the effects are because of the telemonitoring intervention. In a longitudinal study that evaluated the change in HbA_1c_ values after the usual approach to start insulin therapy in 779 patients in primary care practices in Germany and 646 patients in the United Kingdom, with a mean HbA_1c_ of 8.1% (SD 1.3%) and 9.3% (SD 1.5%), respectively [33], the average-adjusted HbA_1c_ improvements in the first 12 months were 0.5% (95% CI 0.4%-0.6%) in Germany and 1.0% (95% CI 0.7%-1.3%) in the United Kingdom. Between 12 and 36 months, these improvements in glycemic control were maintained in both patient groups, without additional improvement in glycemic control. This would suggest that using telemonitoring to initiate insulin is more effective, and in our group, the reduction in mean HbA_1c_ was maintained when we audited the group data in 2019. A randomized controlled trial (RCT) of telemedicine with lifestyle adjustment and titration of metformin plus other oral hypoglycemics or insulin in a Danish type 2 diabetes population demonstrated a 14.6% drop in HbA_1c_ (−15 mmol) with telemedicine and 10.6% (−10 mmol) reduction in their control group [34]. In our retrospective audit of 12 patients commencing insulin in 2015, we saw no clinically significant change in HbA_1c_, which may be because of the multiple barriers to optimal insulin treatment in primary care at the patient and practitioner levels, and this demonstrates the need for structured self-management support and integrated support systems [4].

### Adverse Effects

Some patients did experience hypoglycemia both during and after the intervention, indicating the need for monitoring and support for this population. In an RCT aimed at reducing cardiovascular events in patients with type 2 diabetes using intensive therapy vs standards therapy to reduce HbA_1c_, the rate of hypoglycemic episodes requiring medical assistance was 3.1% in the intensive therapy group and 1.0% in the standard therapy group, and the mean weight gain at 3 years was 3.5 kg and 0.4 kg in the 2 groups, respectively [35]. In our study, no significant change occurred in weight over the 6-month period. Hypoglycemia and weight gain are among the many recognized worries experienced by both patients and health care practitioners when considering insulin treatment [36]. Although 13% (5/39) of patients had episodes of hypoglycemia, both the patients and the CNS participants felt that telemonitoring increased efficacy and patient safety in the transition to insulin therapy. The intervention allowed more timely adjustment of insulin levels, which leads to faster control of blood sugar levels. Patients reported a reduced need to visit the GP and hospital clinics probably because of the increased level of contact with the CNS during the intervention.

### Acceptability

In our study, the use of telemonitoring while transitioning to insulin therapy was highly acceptable to patients, and the use of the technology caused them few problems. There were minor issues with uploading results at the start of the intervention, which could be overcome with an additional visit or better scheduling of the visit from the technology support person.

This intervention has the potential to allow the CNS team to provide more efficient and safe care to the patients, a finding corroborated by Jalil et al [37], but it comes at a cost. The telemonitoring system generated a large dataset for each patient, which had to be reviewed. On reflection, the team felt that they should have availed of the full track, trend, and triage service that is offered as part of the telemonitoring system. This has been found in other studies with one exasperated GP referring to the management of the *tsunami* of patient monitoring data generated by the Whole System Demonstrator project of telemonitoring in long-term conditions in the United Kingdom [38].

The increase in the workload of the team could have been alleviated by administrative support. This would enhance the service by freeing the CNS team to focus on their health care role. There were some teething issues with the introduction of the technology, which caused frustration in both patients and the CNS team. The learning that occurred during the project would allow for a more streamlined approach to the introduction of telemonitoring, with several recommendations for a change to integrating the system with the current service. Although our CNS sample size was small, the findings mirror those of larger studies that have suggested that if the telemonitoring system is not streamlined with the current models of practice, this will impede their uptake by health care professionals [30,38].

### Conclusions

The use of telemonitoring while transitioning to insulin therapy was highly acceptable to patients with high satisfaction and increases in confidence and knowledge. The intervention transformed the nurse-patient relationship and resulted in the empowerment of patients to self-manage. Patients reported an increased awareness and level of responsibility and confidence for self-management of diabetes. There was a significant decrease in the DDS score, and both patients and the CNS team reported reduced patient distress. Some patients also expressed confidence in their knowledge and skill to self-manage their insulin dosage. The CNS team also indicated an increase in their confidence to facilitate patients to self-manage. There was a positive impact on the nurse-patient relationship, with a balancing out of power and a sense of letting go from the CNS team. The increased sense of partnership was empowering to both parties.

There was a clinically and statistically significant drop in HbA_1c_, as expected with the introduction of insulin in glucose management, with no significant impact on weight and improved sense of patient safety from the patient and practitioner perspective.

Overall, the use of the technology caused a few minor problems for patients. The intervention allowed the CNS team to provide more efficient and safe care to the patients, but it came at a cost. There was a significant increase in the administrative workload of the team, which could have been alleviated by administrative support. There were some teething issues with the introduction of the technology, which caused some frustration in both patients and the CNS team, which may be alleviated with a more streamlined approach and integrating the system with the current service.

### Limitations and Further Research

Some limitations of this study need to be considered, and where appropriate, recommendations for further research are proposed. Data in this study were generated through convenience sampling of patients attending one health care region in Ireland, and the number of patients recruited was restricted to 40 by limited nursing and technological resources. In addition, as this was a volunteer sample, these participants may be more motivated toward successful self-management of their diabetes and transition to insulin. Considering this, the findings of this study cannot be generalized to other settings.

The small sample size of both the patient and the CNS groups results in very low study power, which reduces reliability and generalizability. This study design did not allow comparison of this intervention with a control group, which limits the ability to fully determine the effectiveness of the intervention. We audited a retrospective cohort of patients to explore the impact of the intervention; however, we recognize the methodological limitations of this approach. A larger RCT would be useful to allow causal comparisons to be made between telemonitoring-supported care of type 2 diabetes during transition to insulin and conventional care. Measurement of other performance metrics such as frequency of GP visits, levels of technical support needed, and time spent by CNSs in telephone support would also determine the feasibility of such an approach. However, the mixed methods approach used in this study, drawing on both quantitative and qualitative data to support findings, does demonstrate congruence toward patient empowerment experiences when using telemonitoring with telephone support to self-manage diabetes.

Future research design in this area might also consider examining whether improvements in patient outcomes are associated with the effects of using the monitor alone or in combination with increased telephone support and/or the use of an insulin titration scale. Moreover, although this study did look at T1 data and examined the effect at the end of the 12-week intervention and at 3-month follow-up and found positive effects in relation to decreased DDS scores and increased DES scores, further research that would examine if these positive effects were sustained over a longer period would lend strength to the value of such interventions.

## Appendix

Multimedia Appendix 1 Participant experiences themes and subthemes.

Multimedia Appendix 2 Clinical nurse specialist experiences themes and subthemes.

## Abbreviations

CNS: clinical nurse specialist
DDS: diabetes distress scale
DES: diabetes empowerment scale
eHealth: electronic health
GP: general practitioner
HbA_1c_: hemoglobin A_1c_
HSE: Health Service Executive
MD: mean difference
RCT: randomized controlled trial
T1: baseline
T2: end of the intervention
T3: 3 months postintervention
