# Supplementary material for: Patient Empowerment Using Electronic Telemonitoring With Telephone Support in the Transition to Insulin Therapy in Adults With Type 2 Diabetes: Observational, Pre-Post, Mixed Methods Study
Source: J Med Internet Res. 2020 May 14;22(5):e16161. doi: 10.2196/16161 (PMC7256748; doi:10.2196/16161)
Supplement: Multimedia Appendix 1 [file jmir_v22i5e16161_app1.docx]

*Table 2 Participant Experiences Themes and Sub Themes*

| **Theme** | **Associated sub themes** |
| --- | --- |
| **Psychological impact of diabetes** | Anger, agitation, stress,  Blame - self and family members, education system, government  Depression  Distress |
| **Diabetes empowerment** | **Changed mindset**   - Complacent in the past - Increased awareness of impact of choices and lifestyle - Being accountable - Extra scrutiny from hcp   **Learning to manage diabetes**   - New knowledge about health behaviour - Nutrition and blood glucose - Sugar content of food and drinks - Sleep and blood glucose - How to use the insulin adjustment tool   **Gaining control and increased confidence**   - Reduced distress - Support and encouragement - Taking action and seeing good results - Mastering insulin adjustment - Suggestions for networking with peers online for social support |
| **CNS Support (Nurse in the corner)** | **Security, comfort, safety**   - Vulnerability while commencing insulin - Close monitoring with professional oversight provided safety - Gives security and comfort   **Quick intervention**   - Quick adjustment of insulin to achieve glycaemic control - *It’s like having your own diabetic nurse every day*   **Reduced hospital and GP visits**   - Fewer attendances at the GP or hospital - Potential savings for the healthcare system   **Feelings about ending telemonitoring**   - Mixed feelings about ending the technology - Concerns about depending on telemonitoring - Worries about returning to previous behaviours – not monitoring blood glucose manually - Reduced confidence without telemonitoring |
| **Using the technology** | Minor problems with uploading data  Timing of training session –on day of first upload  Keeping machine switched on  Well supported with issues  Further hub development to make user friendly and include mobile app |
