# Supplementary material for: Patient Empowerment Using Electronic Telemonitoring With Telephone Support in the Transition to Insulin Therapy in Adults With Type 2 Diabetes: Observational, Pre-Post, Mixed Methods Study
Source: J Med Internet Res. 2020 May 14;22(5):e16161. doi: 10.2196/16161 (PMC7256748; doi:10.2196/16161)
Supplement: Multimedia Appendix 2 [file jmir_v22i5e16161_app2.docx]

*Table 3 CNS Experiences Themes and Sub Themes*

| **Theme** | **Associated sub themes** |
| --- | --- |
| **Usual transition to insulin** | Busy clinics, limited time to inform and educate patients about insulin therapy  Follow-up calls to a ‘new-to-insulin’ patient difficulties – timing, reliance on patient to make contact with problems, long time to achieve optimal insulin dose, unsafe to rely on patients to disclose relevant and complete information about their blood sugar control over the phone |
| **Safe transition to insulin therapy using telemonitoring** | **Access to accurate and complete results**;   - Clearer picture of the patients’ blood sugar profiles and the effectiveness of their treatment regime - Data all in one place on the web-based platform - Access to data remotely on tablets   **Advising and managing appropriately and safely.**   - More focused and tailored discussions with patients in regards to their blood sugar and insulin management - Safer patient management because of access to accurate data - Picking up and following up hypoglycaemic event that patients did not report verbally - Reduced need for patients to travel to centres/clinics |
| **Patient empowerment** | **Increased patient knowledge and confidence**   - Confidence in self-managing insulin - Less stress and worry about diabetes and changes to treatment - Reassured by being monitored or watched or supervised   **Taking control**   - Increasing levels of responsibility for self-managing their condition - Independently and appropriately adjusting their own insulin levels - Motivation to self-manage related to seeing results and CNS seeing blood glucose levels   **Using the insulin self-adjustment tool**   - Promoted self-adjustment safely - Selected patients that they assessed and deemed suitable for using the tool - Patient confidence in self-management of insulin adjustment increased - CNS had feared that the tool could be used incorrectly and put patients at risk - on reflection would have used it with a lot more patients - Didn’t really have hypos as a result of using the tool - Tool would address problem of insulin not being increased quickly enough   **Enhanced nurse-patient partnership/relationship**   - enhanced partnership; - equal footing in goal achievement. - Equal relationship - easing the nurse into the watchful observer role - teamwork |
| **Administrative supports and requirements** | Need adequate resources to support such a system  Larger volume of patient data for review  Nursing resource for timely patient data review – patient safety concern  Administrative support –electronic referrals for telemonitoring, informing GPs, scanning and uploading patient data to the web platform  *ICT –* Telehealth system needs to be integrated with current patient information system |
| **Technology** | **Easy to Use**   - Simplicity and ease allowed use by all age groups including older patients - Skip button on this system caused a lot of frustration   **Technology Preparation**   - CNS need to see fully installed live telemonitoring hub box in a patient home to help troubleshoot patient problems - Family members and the FOLD hub installer were very good sources of technical support and advice - Need detailed visual operating instructions for patients   **Technology Problems**   - Need reliable, mobile and fast access to the telemonitoring web platform - Technology and technical support needs to be integrated within the current health information system - Delays with installation particularly in holiday periods - Teething problems -CNS needs direct contact with company installing the telemonitoring hub; need reliable timely contact from company when results failed to upload - Regretted not availing of the full track, trend and triage service as part of the telemonitoring system |
